# Is There Really Relationship between Androgenetic Alopecia and Metabolic Syndrome?

**DOI:** 10.1155/2015/980310

**Published:** 2015-11-04

**Authors:** Seyran Ozbas Gok, Asli Akin Belli, Emine Dervis

**Affiliations:** ^1^Department of Dermatology, Haseki Training and Research Hospital, 34080 Istanbul, Turkey; ^2^Department of Dermatology, Mugla Sitki Kocman University Training and Research Hospital, 48000 Mugla, Turkey

## Abstract

*Background.* There are several studies investigating the relationship between androgenetic alopecia (AGA) and metabolic syndrome (MS) with conflicting results. *Objective.* We sought to investigate whether there is a relationship between AGA and MS. *Methods.* A case-control study including 74 male patients with AGA and 42 male controls was conducted. Age, duration of AGA, AGA onset age, anthropometric measures, body mass index, lipid parameters, fasting blood glucose, blood pressure, and presence of MS were recorded. *Results.* Of the 74 male AGA patients (age range 20–50 years, mean 32.14), 24 were in stage 2, 26 were in stage 3, 17 were in stage 3V, 1 was in stage 5, and 6 were in stage 7. There was no significant difference in the rate of MS between AGA and control groups (*P* = 0.135). Among the evaluated parameters, only systolic blood pressure in AGA group was significantly higher than control group. *Conclusion.* In contrast to the most of the previous studies, our study does not support the link between AGA and MS. To exclude confounding factors such as advanced age and therefore metabolic disturbances, further studies are needed with large group of AGA patients including different age groups and varying severity.

## 1. Introduction

Androgenetic alopecia (AGA) is a hair loss in specific patterns depending on circulating androgens in genetically predisposed men and women. Androgenetic alopecia affects 80% of men by the age of 70 years. In women, the incidence of AGA is 2–5% by the age of 50 and the ratio rises to 40% at age of 70. Although the main etiological factors are the same, phenotypic manifestations are different in men and women. Men usually have bitemporal and vertex hair loss leading to the complete baldness. In women, frontal hairline is usually preserved and complete baldness does not occur [1].

Metabolic syndrome (MS) is a group of metabolic disorders such as glucose intolerance, insulin resistance (IR), central obesity, dyslipidemia, and hypertension associated with increased risk of cardiovascular disease [2]. There are several studies investigating the relationship between AGA and MS, as well as IR with conflicting results [3–8]. In this study, we aimed to investigate whether there is a relationship between AGA and MS.

## 2. Patients and Methods

Between August 2012 and January 2013, a case-control study including 74 patients with AGA and 42 male controls was conducted in the Dermatology Outpatient Clinic of Haseki Training and Research Hospital, Istanbul. Ethic committee approval was obtained prior to the study. Inclusion criteria for patients with AGA were as follows:

1. Being between 20 and 50 years of age.
2. Absence of a known glucose metabolism disease.
3. Absence of a known coronary artery disease.
4. No history of drug use that affects carbohydrates metabolism (especially glucocorticoids).

The control group included age- and gender-matched male patients who have the inclusion criteria and admitted to the outpatient clinic with various dermatological complaints except alopecia.

AGA group included the patients who admitted to our outpatient clinic for alopecia or any dermatological problems that meet the inclusion criteria. Diagnosis of AGA was based on characteristic clinical findings. For grading AGA; Hamilton-Norwood classification was used in the patient group [1, 9].

Age, anthropometric measures (weight, height, and waist circumference), body mass index (BMI), lipid parameters (triglyceride, total cholesterol, high density lipoprotein, and low density lipoprotein), fasting blood glucose (FBG), blood pressure values of the participants' were evaluated. Waist circumference (WC) levels of >94 cm were recommended as cutoff point for abdominal obesity. BMI was calculated using the formula weight (kg)/height (m^2^). Lipid parameters and FBG levels were studied after a 12-hour fasting period. Triglyceride level >150 mg/dL and HDL level <40 mg/dL were recommended as cutoff for dyslipidemia. FBG level ≥100 mg/dL was recommended as cutoff for impaired fasting glycemia. Systolic BP ≥135 mmHg and diastolic BP ≥85 mmHg were recommended as cutoff points for hypertension. The onset age, the duration of AGA, smoking history, and sports life were also recorded. The onset age of ≤35 years was recommended as early-onset AGA.

Based on the diagnostic criteria of International Diabetes Federation (IDF-2005), waist circumference >94 cm and at least two of the following criteria, triglyceride value >150 mg/dL or specific treatment for this lipid abnormality, high density lipoprotein <40 mg/dL or specific treatment for this lipid abnormality, blood pressure ≥130/85 mmHg or antihypertensive treatment, and fasting blood glucose ≥100 mg/dL or diagnosed diabetes mellitus, were accepted as MS [10].

In all patients, complete blood count, serum iron, total iron binding capacity, serum ferritin, free T3, free T4, and thyroid stimulating hormone (TSH) were performed to determine possible iron deficiency anemia and thyroid disorders.

For the data analysis, the statistical program “SPSS for windows 21.0” was employed. Mean, standard deviation, ratio, and frequency were used for descriptive statistics of the data. The distribution of variables was checked with Kolmogorov-Smirnov test. Independent samples *t*-test and Mann-Whitney *u* test were used for the analysis of quantitative data. The chi-square test was used for the analysis of categorical data. *P* < 0.05 was assessed as significant.

## 3. Results

Seventy four male AGA patients (age range 20–50 years, mean 32.14) and 42 controls (age range 20–50 years, mean 34.40) were investigated. Of 74 AGA patients 24 (32.4%) were in stage 2, 26 (35.1%) were in stage 3, 17 (23%) were in stage 3V, 1 (1.4%) was in stage 5, and 6 (8.1%) were in stage 7 according to Hamilton-Norwood classification.

In AGA group, there were no significant differences in the participants' age, weight, height, BMI, sport life, TG, total cholesterol, HDL, LDL, and diastolic pressure values (*P* > 0.05). AGA group had significantly high systolic blood pressure levels and control group had significantly high FBG and waist circumference levels (*P* < 0.05) (Table 1). There was no significant difference in the rate of MS between AGA and control groups (Table 2) (*P* > 0.135).

In AGA group, the onset age of AGA and the duration of AGA were not significantly different in the patients with or without MS (*P* > 0.05).

When patients with stages I–IV were assessed as mild-moderate and patients with stages V–VII were assessed as severe, there was no significant difference between the patients with or without MS (*P* < 0.05).

## 4. Discussion

There are several studies investigating AGA and metabolic disorders related to MS with conflicting results [3–8]. Although some studies have reported a relationship between AGA and MS, pathophysiological connection between these two diseases has not been completely elucidated [11].

Metabolic syndrome is a group of metabolic disorders associated with increased risk of cardiovascular disease [2]. In the pathogenesis of MS, several factors such as genetic predisposition, insulin resistance (IR), obesity, hypertension, dyslipidemia, vascular abnormalities, inflammation, hyperandrogenism, uric acid deficiency, and vitamin D deficiency are considered to be responsible. In a study performed by Acibucu et al., the rate of IR and MS was significantly high in 80 early AGA patients [3]. The link between AGA and MS has not been clarified yet. However, two hypotheses have been proposed that, firstly, both of AGA and cardiovascular diseases are related to excess of androgens and secondly, hormonal changes such as hyperandrogenism may play a role in the development of AGA and hypertension [12]. Mumcuoglu et al. found a relationship between AGA and IR but not with MS in 50 male patients with grade ≥3 AGA [4]. The action mechanism of insulin on the AGA is not clear. It has been suggested that insulin may be an important factor in the pathogenesis of AGA by causing vasoconstriction and nutrient deficiency. It may also be effective by enhancing the effects of testosterone [5]. Insulin and insulin-like growth factor-1 may enhance DHT levels by inducing 5-*α*-reductase activity in obese patients [13]. Matilainen et al. noted that IR may be a pathophysiological mechanism or enhancing factor in early AGA [6]. In another study performed by Matilainen et al., some parameters associated with IR were significantly high in 342 female AGA patients [7]. In our study, insulin levels and insulin resistance were not evaluated.

In contrast to the other studies, Nabaie et al. did not find a relationship between AGA and the parameters, FBG, serum fasting insulin levels, total cholesterol, TG, HDL, and IR in 97 male AGA patients [8]. Similarly, we did not find a positive correlation between AGA and MS. Only systolic blood pressure levels were significantly higher than control group. Although controls were selected metabolically normal subjects, FBG and mean waist circumference levels were significantly higher in the control group. These unexpected results were attributed to be incidental. Additionally, there was no relationship between the AGA onset age and the duration of AGA and MS. Positive relation of AGA with MS and IR reported in the previous studies may be due to the confounding factors that the patients had advanced age and thus metabolic abnormalities could be plausible. The study reported by Giltay et al. also supports our results. In this study, 81 female-to-male transsexuals treated with testosterone esters had male-pattern baldness without any cardiovascular disease risk factors such as increased blood pressure, levels of lipid, and insulin. It was noted that male-pattern alopecia does not indicate increased cardiovascular disease risk [14]. In another study involving 60 AGA patients with or without MS, the patients with MS were likely to have IR rather than the patients without MS, and true association between AGA and IR has been denied [5].

The relationship between early AGA and serious cardiovascular events such as myocardial infarction (MI) and fatal ischemic heart disease has been shown, but explaining mechanisms are not clear [6]. Androgen receptors have been found in the arterial wall endothelium but have not clarified whether there are direct effects [15]. Lesko et al. investigated 665 male AGA patients and 772 control patients who had acute myocardial infarction under 55 years of age; they found higher rates of AGA on the vertex [16]. In terms of cardiovascular risk factors, systolic blood pressure levels were significantly higher in our AGA group. There are some studies reporting an association between hypertension, as well as aldosterone levels and AGA. It has been proposed that specific mineralocorticoid receptor antagonists could be used in the treatment of AGA [17, 18].

## 5. Conclusions

Even though some studies undetected a relationship between AGA and MS, it has been found in many and has emphasized the importance of early diagnosis. In contrast to previous reports, we did not find a relationship between AGA and MS. Although there was no correlation between AGA and MS, to detect an increase in systolic blood pressure levels may be remarkable in terms of being a cardiovascular disease risk factor. Moreover, this low rate of MS may be related to the fact that our study did not include elderly patients and majority of our patients had mild alopecia compared to the other studies. On this topic, there is a need of large group of AGA patients including different age groups and varying severity.

## Figures and Tables

**Table 1:** Comparison of MS parameters and BMI in AGA and control groups.

	Patients (*n* = 74) *n* (%)	Controls (*n* = 42) *n* (%)	*P*
BMI ≥25 kg/m^2^	35 (47.2)	26 (61.9)	0.130
FBG ≥100 mg/dL	1 (1.3)	9 (21.4)	**<0.001**
TG ≥150 mg/dL	23 (31)	17 (40.4)	0.306
HDL <40 mg/dL	28 (37.8)	17 (40.4)	0.779
Systolic BP ≥135 mmHg	16 (21.6)	3 (7.1)	**0.043**
Diastolic BP ≥85 mmHg	5 (6.7)	1 (2.3)	0.415
Waist C >94 cm	23 (31.1)	21 (50)	**0.044**

Chi-square test. MS: metabolic syndrome; AGA: androgenetic alopecia; BMI: body mass index; FBG: fasting blood glucose; TG: triglyceride; HDL: high density lipoprotein; BP: blood pressure; Waist C: waist circumference.

**Table 2:** The rate of metabolic syndrome in AGA and control groups.

	AGA group		Control group		*P*
	*n*	%	*n*	%	
Metabolic syndrome					
Present	11	14.9%	11	26.2%	0.135
Absent	63	85.1%	31	73.8%	

Chi-square test. AGA: androgenetic alopecia.

## References

[1] Paus R., Olsen E. A., Messenger A. G., Wolff K., Goldsmith L. A., Katz S. I., Gilchrest B. A., Paller A. S., Leffell D. J. (2008). Hair growth disorders. *Fitzpatrick’s Dermatology in General Medicine*.

[2] Fulop T., Tessier D., Carpentier A. (2006). The metabolic syndrome. *Pathologie Biologie*.

[3] Acibucu F., Kayatas M., Candan F. (2010). The association of insulin resistance and metabolic syndrome in early androgenetic alopecia. *Singapore Medical Journal*.

[4] Mumcuoglu C., Ekmekci T. R., Ucak S. (2011). The investigation of insulin resistance and metabolic syndrome in male patients with early-onset androgenetic alopecia. *European Journal of Dermatology*.

[5] Abdel Fattah N. S. A., Darwish Y. W. (2011). Androgenetic alopecia and insulin resistance: are they truly associated?. *International Journal of Dermatology*.

[6] Matilainen V., Koskela P., Keinänen-Kiukaanniemi S. (2000). Early androgenetic alopecia as a marker of insulin resistance. *The Lancet*.

[7] Matilainen V., Laakso M., Hirsso P., Koskela P., Rajala U., Keinänen-Kiukaanniemi S. (2003). Hair loss, insulin resistance, and heredity in middle-aged women. A population-based study. *Journal of Cardiovascular Risk*.

[8] Nabaie L., Kavand S., Robati R. M. (2009). Androgenic alopecia and insulin resistance: are they really related?. *Clinical and Experimental Dermatology*.

[9] Olsen E. A. (2001). Female pattern hair loss. *Journal of the American Academy of Dermatology*.

[10] Alberti K. G., Zimmet P., Shaw J. (2005). The metabolic syndrome—a new worldwide definition. *The Lancet*.

[11] Arslan M. (2006). Metabolik sendrom: tanımı, patogenezi, tanı kriterleri ve bileşenleri. *Turkiye Klinikleri Journal of Internal Medical Sciences*.

[12] Trieu N., Eslick G. D. (2014). Alopecia and its association with coronary heart disease and cardiovascular risk factors: a meta-analysis. *International Journal of Cardiology*.

[13] Yang C.-C., Hsieh F.-N., Lin L.-Y., Hsu C.-K., Sheu H.-M., Chen W. (2014). Higher body mass index is associated with greater severity of alopecia in men with male-pattern androgenetic alopecia in Taiwan: a cross-sectional study. *Journal of the American Academy of Dermatology*.

[14] Giltay E. J., Toorians A. W. F. T., Sarabjitsingh A. R., de Vries N. A., Gooren L. J. G. (2004). Established risk factors for coronary heart disease are unrelated to androgen-induced baldness in female-to-male transsexuals. *Journal of Endocrinology*.

[15] Pinkney J. H., Stehouwer C. D. A., Coppack S. W., Yudkin J. S. (1997). Endothelial dysfunction: cause of the insulin resistance syndrome. *Diabetes*.

[16] Lesko S. M., Rosenberg L., Shapiro S. (1993). A case-control study of baldness in relation to myocardial infarction in men. *The Journal of the American Medical Association*.

[17] Ahouansou S., Le Toumelin P., Crickx B., Descamps V. (2007). Association of androgenetic alopecia and hypertension. *European Journal of Dermatology*.

[18] Arias-Santiago S., Gutiérrez-Salmerón M. T., Buendía-Eisman A., Girón-Prieto M. S., Naranjo-Sintes R. (2010). Hypertension and aldosterone levels in women with early-onset androgenetic alopecia. *British Journal of Dermatology*.

